# Curcumin as a Therapeutic Option in Retinal Diseases

**DOI:** 10.3390/antiox9010048

**Published:** 2020-01-06

**Authors:** Daniel López-Malo, Carlos Alberto Villarón-Casares, Jorge Alarcón-Jiménez, Maria Miranda, Manuel Díaz-Llopis, Francisco J. Romero, Vincent M. Villar

**Affiliations:** 1 Facultad de Ciencias de la Salud, Universidad Europea de Valencia, 46010 Valencia, Spain; daniel.lopez2@universidadeuropea.es (D.L.-M.); carlosalberto.villaron@universidadeuropea.es (C.A.V.-C.); 2 Facultad de Medicina y Ciencias de la Salud, Universidad Católica de Valencia, 46001 Valencia, Spain; jorge.alarcon@ucv.es; 3 Facultad de Ciencias de la Salud, Universidad CEU Cardenal Herrera, 46315 Moncada, Spain; mmiranda@uchceu.es (M.M.); vmvillar@uchceu.es (V.M.V.); 4 Facultad de Medicina y Odontología, Universitat de Valencia, 46010 Valencia, Spain; manuel.diaz@uv.es; 5 Hospital General de Requena, Conselleria de Sanitat, Generalitat Valenciana, 46340 Valencia, Spain

**Keywords:** curcumin, oxidative stress, retina, retinal diseases

## Abstract

The retina is subjected to oxidative stress due to its high vascularization, long time light exposition and a high density of mitochondria. Oxidative stress can lead to pathological processes, like cell apoptosis, angiogenesis and inflammation ending in retinal pathologies. Curcumin, a major bioactive component obtained from the spice turmeric (*Curcuma longa*) rhizome has been used for centuries in Asian countries for cooking and for curing all kinds of diseases like dysentery, chest congestion and pain in general, due to its antioxidant effects. Curcumin prevents the formation of reactive oxygen species and so it is a good protective agent. Curcumin has shown also anti-inflammatory, and antitumor properties. Curcumin is a natural product, which can be a therapeutic option in a variety of retinal diseases due to its pleiotropic properties. Some drawbacks are its poor solubility, bioavailability and lack of stability at physiological conditions; which have been shown in curcumin skeptical publications. In this review, we provide some lights and shadows on curcumin administration on the major retinal pathologies.

## 1. Introduction

The retina, located in the posterior segment of the ocular globe, in contact with the vitreous humor and the retinal pigment epithelium (RPE) and anteriorly with the ciliary epithelium of the pars plana. A structure present in the eye of most vertebrates and some mollusks, which embryonically outgrows, with the optic nerve, from the diencephalon. In relation to this fact, the retina is regarded as part of the central nervous system (CNS). The retina has several cell types, among them there are two types of photoreceptors: cones and rods. Cones, located in the macula lutea, detect the fine shape, colors and the motion of objects. Cones require high intensity of light and work in photopic light conditions (>10 lux). Rods, placed at the periphery, lack the color discrimination feature but are much more sensitive to dim light, hence rods work in scotopic light conditions (<0.1 lux, night vision). Half of the neurons that form the optic nerve, retinal ganglion cells (RGCs) are in this region, where bipolar cells, horizontal cells, amacrine cells and Müller cells can also be found [1].

In the retina, photoreceptors and RGCs are particularly vulnerable to oxidative stress due to the environment of high oxygen, glucose oxidation and lipid polyunsaturated fat (PUFA) content, coupled with photo-transduction, leading to increased reactive oxygen species (ROS) production [2]. It has been observed that ROS imbalance is involved in many retinal diseases. Among the primary reactive species are superoxide anion, singlet oxygen, hydroxyl radical, hydrogen peroxide (H_2_O_2_), peroxynitrite or nitric oxide. While the oxidation of PUFA can lead to the formation of malondialdehyde (MDA) and 4-hydroxynonenal (4-HNE) [3]. All these ROS can vanquish the endogenous antioxidant enzymes, like superoxide dismutase (SOD), glutathione peroxidase (GPx) and catalase (CAT) [4].

In most of the retinal pathological processes like angiogenesis, apoptosis or inflammation, ROS play an important role. Subsequently, numerous exogenous antioxidant compounds have been used in the literature to avoid the effects of ROS. Different natural compounds stand out as potential nutraceuticals, such as, docosahexaenoic acid [5], carotenoids like lutein [6] and zeaxanthin [7]; saffron [8,9], catechins [10] and ginkgo biloba extract [11]. Especially curcumin has drawn researcher’s attention during the last years [12,13,14,15,16].

**Figure 1:**
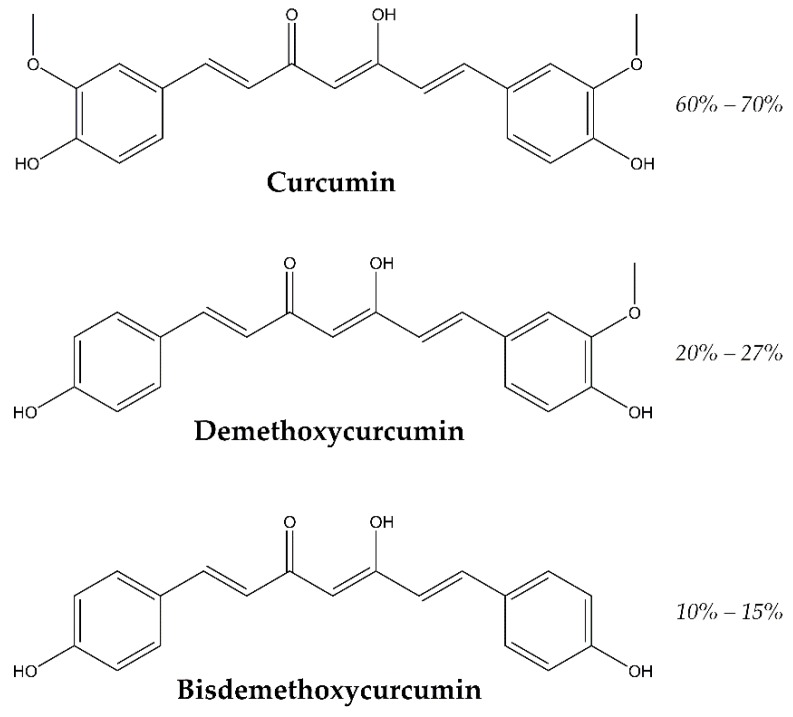
Structure and relative abundance of major curcuminoids.

Turmeric is the curcuma longa’s rhizome, which has curcuminoids as curcumin and mono-demethoxycurcumin and bis-demethoxycurcumin, shown in Figure 1, and also sesquiterpenoids as curcumenes and turmerones. All these derivatives have been identified by capillary GC–MS (gas chromatography–mass spectrometry) and HPLC (high performance liquid chromatography) analysis in other species of curcuma as well [17].

Curcumin also known as diferuloylmethane, E100 or Natural Yellow 3 (IUPAC name is (1E, 6E)-1,7-bis(4-hydroxy-3-methoxyphenyl)-1,6-heptadiene-3,5-dione) is an orange-yellow solid at room temperature and its chemical formula C_21_H_20_O_6_ (MW 368.38 g/mol). Curcumin has two aromatic ring systems containing o-methoxy phenolic groups, which are linked with a seven-carbon linker consisting of an α-, β- unsaturated β-diketone moiety.

Curcumin exists in two tautomeric forms, the keto–enol and di-keto tautomers. The predominant tautomer of curcumin is the keto–enol form when it is present in polar organic solvents. This tautomer possesses intramolecular hydrogen bonding in the keto–enol moiety and π-conjugation is kept across the molecule, which results in an ultraviolet-visible (UV-Vis) absorption peak around 420 nm [18]. Curcumin is a hydrophobic molecule being insoluble in water, only approximately 30 nM, and readily soluble in polar solvents such as methanol, ethanol, acetonitrile, chloroform, dimethyl sulfoxide (DMSO) and ethyl acetate. Curcumin has high potential to scavenge reactive oxygen species, which makes it an important therapeutic and antioxidant molecule [19]. Most free radical oxidants participate in electron transfer reactions and hydrogen abstraction. Reports suggest that during free radical reactions with curcumin, the hydrogen of phenol-OH group is readily abstractable, producing phenoxyl radicals, which are resonance stabilized across the keto–enol structure [20].

## 2. Diabetic Retinopathy (DR)

One of the main secondary complications of type 1 and type 2 diabetes is diabetic retinopathy [21]. Diabetic retinopathy (DR) has associated retina edema, hemorrhage, ischemia, microaneurysms, augmented neovascularization and neuronal degeneration in the retina [22]. The progress of this disorder alters the photoreceptors and the blood vessels of the retina. The retina has a high content in polyunsaturated fatty acids (PUFA) and a high oxygen and glucose uptake compared with other tissues, making the retina more prone to oxidative stress. It has been demonstrated that oxidative stress not only renders DR condition, but even after the glycemic levels are back to homeostatic ones it hinders the remission of the DR. Several metabolic pathways are involved in the reactive oxygen species imbalance: polyol pathway, hexosamine pathway, advanced glycation end product (AGE) pathway and protein kinase C (PCK) pathway [23]. One of the main angiogenic factors implicated in ocular neovascularization is vascular endothelial growth factor (VEGF) [24]. Another investigation led by Chiu described the activation of nuclear enzyme poly(ADP-ribose) polymerase (PARP) after an oxidative insult in PARP^−/−^ mice and diabetic rats; and how it is related to increase in extracellular matrix protein expression and its direct relation with the activation of transcription factors as nuclear factor kappa-light-chain-enhancer of activated B cells (NFκB) and extracellular matrix proteins by upregulating endothelin-1 (ET-1) [25].

There are studies on streptozotocin-induced diabetic rat that describe that the use curcumin decreases oxidative stress and prevented the loss of the chaperone function of alpha-crystallin [26], other authors describe the inhibition of VEGF expression in retina under hyperglycemic conditions [27]. In the same year Kowluru and Kanwar evaluated the total antioxidant capacity and the levels of glutathione (GSH), oxidatively modified DNA (8-OHdG), nitrotyrosine, interleukin 1 beta (IL-1β), NFκB and VEGF in Lewis streptozotocin-induced diabetic rats after the administration of curcumin (0.5 g/Kg) during six weeks; total antioxidant capacity was restored, GSH levels were partly restored and the levels of inflammatory markers were diminished when compared with matched weight controls [28]. In the same line, Bharara et al. claimed that glucose induced upregulation of DNA excision repair protein (ERCC1) and DNA repair endonuclease XPF (ERCC4) lead to a rise in fibronectin production via p300-dependent pathway in endothelial cells, retina and kidney of streptozotocin-induced diabetic rats [29].

Curcumin has also been investigated in vitro on human retinal pigment epithelial immortalized cell line derived from Amy Aotaki-Keen eyes (ARPE-19), human retinal endothelial cells (HRECs) and human retinal pericytes (HRPCs) and in vivo on male New Zealand white rabbits. Showing that curcumin administration protected ARPE-19 cells from H_2_O_2_ oxidative damage in a dose-dependent manner, being statistically significant at 100 µM. Regarding HERCs cells, treatment with 10 µM curcumin reverted the effect of 40 mM glucose leading to a significant decrease (*p* < 0.01) of tumor necrosis factor (TNF), similar to control cells, and on HRPCs cells the use of 10 µM curcumin prevented cell death caused by 40 mM glucose. On the in vivo test, different commercial available products containing curcumin were assessed, namely, curcumin formulation with a polyvinylpyrrolidone-hydrophilic carrier, curcumin-phosphatidylcholine complex and curcumin + piperine, after the intake at different times, the animals were sacrificed and the retinae were analyzed by HPLC-MS/MS showing that just the formulation with polyvinylpyrrolidone-hydrophilic carrier was able to reach the retina with a t_MAX_ of 6 h after oral administration [30].

Bucolo et al. studied the protective effect of curcumin against high glucose insult on ARPE-19 cells. The administration of curcumin improved the cell viability in a dose dependent manner, significantly using 15 and 20 µM concentration of curcumin. The proposed mechanism involves the modulation of extracellular signal–regulated kinases (ERK1/2) pathway, upregulating nuclear respiratory factor 2 (Nrf2) expression and HO-1 activity. It was later confirmed using a mitogen-activated protein kinase (MAPK) inhibitor (PD98059) and the apoptotic cleaved caspase-3 protein [31].

## 3. Eye Antitumor Activity (Retinal and Choroidal Tumors)

The main malignancy located in the retina of children is retinoblastoma [32]. The trigger of this disease is the inactivation of the *RB1* gene, a tumor suppressor gene. The most common early symptom is leukocoria, followed by strabismus. There are multiple treatment options, of which the most used currently is the more eye conservative ones, chemotherapy in situ, in detriment of systemic chemotherapy, radiotherapy and eye enucleation [33]. Yu et al. found than the activation of c-Jun N-terminal kinase (JNK) and p38 MAPK on the retinoblastoma Y79 cell line was induced by curcumin. This finding was corroborated by the use of SP600125 and SB203580, specific inhibitors of JNK and p38 MAPK respectively, which inhibited the apoptosis of the retinoblastoma cell line and suppressed the activation of caspase-9 and -3 [34]. Li et al. studied the effect of curcumin on SO-Rb50 and Y79 cells, retinoblastoma cell lines, and found out that curcumin inhibited cell proliferation, induced apoptosis and inhibited the migratory and invasive capacities of the retinoblastoma cell lines. The authors proposed that curcumin deactivates the Janus kinase-signal transducer and activator of transcription (JAK/STAT) signaling pathway via regulation of microRNA-99, which was supported by the lack of activity of curcumin in microRNA-99a-silenced cells [35]. 

Although not directly generated in the retina, some cases of intraocular lymphoma affect the eye secondarily by metastasis, which tend to migrate to the retinal ganglion cell layer or can be originated within the eye and cause visual diseases [36]. It has been stated in the literature that curcumin possesses anticancer activity, because of the combination of its antioxidant, anti-inflammatory proapoptotic, immunomodulatory and anti-angiogenic properties [37].

Lu et al. studied the growth inhibition effect of curcumin in N18 mouse-rat hybrid retina ganglion cells in vitro, they explained the effect by G2/M phase cell cycle arrest and the induction of apoptosis combined with up-regulation of Bcl-2-associated X protein (BAX) and down regulation of Bcl-2. Curcumin also up-regulated the active form of caspase-8, -9 and -3 suggesting that both mitochondrial and death receptor pathways are implicated by promoting the levels of apoptosis antigen 1 (Fas) and Fas-associated protein with death domain (FADD) [38]. These same authors studying the same cell line concluded that curcumin also inhibited DNA repair genes expression such as *ATM, ATR, BRCA1,* 14-3-3r, DNA-dependent protein kinase (DNA-PK) and O-6-methylguanine-DNA methyltransferase (MGMT) [39]. 

Lin et al. explored the effect of curcumin on the migration and invasion of the same cell line. They observed a dose- and time-dependent protection with best results with a concentration of curcumin of 15 µM administered for 48 h. The authors observed an inhibition in the levels of microRNA (miRNA) in N18 cells, with a decreased expression levels of matrix metalloproteinases (MMPs) MMP-2, MMP-7, focal adhesion kinase (FAK), ras homolog family member A (Rho A) and rho-associated, coiled-coil-containing protein kinase 1 (ROCK1); they also noticed lower levels of growth factor receptor bound protein 2 (GRB2), Ras, protein kinase C (PKC), MKK7, FAK, Rho A, ROCK1, MMP-2, MMP-9, inducible nitric oxide synthase (iNOS), NF-κB p65, Prostaglandin-endoperoxide synthase 2 (COX-2), JNK1/2 and ERK1/2 when curcumin was administered, so they attributed the action of curcumin to an inhibition of MMP-2 and 9 [40].

Burugula et al. studied a murine retinal ganglion cell line (RGC-5) treated with various doses of protein kinase inhibitor staurosporine (SS) and curcumin. Two optimal doses, which were SS (12.5 and 100 nM) or curcumin (2.5 and 100 µM), were injected in C57BL/6 mice. In the in vitro test the results indicated that curcumin diminished SS-mediated cell death at low doses, whereas high doses were toxic; while in vivo a 10 µM dose of curcumin attenuated the protease-mediated death of RGCs and amacrine cells significantly. The authors outlined that curcumin offered a protective effect by restoring NFκB [41].

## 4. Retinal Ischemia-Reperfusion Injury (RIRI)

Retinal ischemia-reperfusion injury (RIRI) is a common pathological process that can result in vision loss. It normally happens as a result of a loss of intraocular perfusion pressure due to ocular hypertension. The factors related to its appearance are inflammatory factor-mediated inflammation, free oxygen radical formation, changes in nitric oxide (NO) formation and calcium cation overload. RGCs are vulnerable to ischemia, leading to the thinning of nerve fiber layer by RGCs injury or loss [42].

Wang et al. found out the protective effect of curcumin on retinal neurons and microvessels against RIRI by the inhibition of NFκB and STAT3 (signal transducer and activator of transcription) and the subsequent overexpression of monocyte chemoattractant protein 1 (MCP-1) on Wistar rats but were unable to reduce the expression of JAK2. Curcumin was used pre and post-RIRI, showing significant protective effects when administered 0.05–0.25 ppm and 0.01–0.25 ppm respectively [43]. Zhang et al. described the reduction of the expression of interleukin-17 (IL-17) and IL-23 in Sprague-Dawley rat retina after RIRI due to the administration of curcumin in a dose dependent manner [44]. Another study claimed the protective effect on the retina of curcumin in stroke spontaneously hypertensive rats (SHR). The protection was observed in retinal neurons and retinal capillaries by the general inhibition of JNK protein expression by curcumin [45]. Mallozzi et al. described the regulatory effect of curcumin on the activity of Ca^2+^/calmodulin-dependent protein kinase II (CAM-KII) on primary Wistar rat retinal cultures and its plausible use against *N*-methyl-d-aspartate receptors (NMDARs) excitotoxicity [46].

## 5. Age-Related Macular Degeneration (AMD)

AMD is a disorder of the central part of the retina—macula—causing progressive impairment of central vision [47]. AMD impact is higher in older people. Its grade of severity is divided in early, intermediate and advanced. Dry and wet phases are distinguished, being the dry phase the most prevalent. The dry phase is characterized by the accumulation of drusen [48], lipoprotein deposits containing beta amyloid precursor in the macula, and the wet phase of AMD is characterized by the proliferation of choroidal neovascularization. The new formed blood vessels are fragile and tend to leak, which allow its content to extravasate and affect vision. It is described that and excess of VEGF induces the wet phase of AMD [49].

Mandal et al. found significant differences in the expression of inflammatory genes in the retinas of curcumin fed Wistar rats (2000 ppm). Suppression of NF-κB was observed and was hypothesized as the source of protection of the retinal cells. The authors also studied the protective effect of curcumin against oxidative stress mediated damage in vitro using retinal cell lines 661W and ARPE-19. A decrease of the expression of early growth response protein 1 (EGR1) and intercellular adhesion molecule 1 (ICAM1) and the induction of the expression of heme oxygenase 1 (HMOX1), thioredoxin (Trx-1) and Nrf2 were found, which supported the protection via Nrf2 transcription factor. Thus, validating curcumin as a nutraceutical compound to prevent or delay AMD [50]. Furthermore, Bhattacharjee et al. studied this effect on human retinal tissues and C8B4 microglial cells culture [51]; Park et al. described the protective effects of the three major curcuminoids at a concentration of 15 μM on ARPE-19 cells after blue light exposure, with a significant reduction in c-Abl and p53 miRNA, which increase the number of apoptotic RPE cells. It is worth to note that the protective effect of curcumin was lost at 20 μM, while still was present for demethoxycurcumin and bis-demethoxycurcumin. It indicated the potential use of curcuma longa extracts as a natural antioxidant to prevent the effects of retinoid-derived ageing pigments, N-retinylidene-N-retinyl-ethanolamine and iso-N-retinylidene-N-retinyl-ethanolamine, which play a significant role in the progression of AMD by generating ROS [52].

## 6. Glaucoma

Glaucoma is one of the major causes of irreversible blindness, with over 60 million people affected worldwide and steadily growing [53,54]. It is a progressive optic neuropathy, characterized by the excavation of the optic nerve, due mainly to high ocular pressure, which leads to a progressive visual field loss by the loss of RGCs [55]. The group of ocular diseases labeled as glaucoma are subdivided in subcategories, being the most prevalent open-angle glaucoma, in which, the aqueous humor does not drain, although the trabecular meshwork is not destroyed and the less frequent closed-angle glaucoma and normal tension glaucoma [56]. Current treatments try to lower the intraocular pressure; and try to restore the RGC population transplanting stem cells, but in many cases the transplanted cells stay undifferentiated [57].

Lin et al. explored the protective effects of curcumin in trabecular meshwork cells (TMCs) from pig eyes; they found that a concentration of 20 μM curcumin was the most efficient to attenuate the effects of oxidative stress on TMCs produced by H_2_O_2_ evidenced by a decrease on the expression of the NF-κB markers, IL-6, endothelial-leukocyte adhesion molecule 1 (ELAM-1), IL-1a and IL-8; and also found a decrease on apoptotic cells was also observed after the H_2_O_2_ insult [58]. More recently, Luo et al. on the same cell line and also using H_2_O_2_ as oxidative stress inducer, found that curcumin activated the nuclear respiratory factor 2 -Kelch like the ECH associated protein 1 (Nrf2-Keap1) signaling pathway in H_2_O_2_ treated TMCs. The authors also noticed that the use of high concentrations of curcumin inhibited not only apoptosis but also necrosis on TMCs [59]; which somehow contradicts another findings reviewed herein, such as the one stated by Park [52]. Furthermore, curcumin has proved to increase viability of RGC in other ocular diseases as described on the RIRI section of this review [53]. Curcumin has also been used to treat glaucoma in form of loaded nanoparticles [60], but this will be discussed later.

## 7. Alzheimer’s Disease, (AD)

Alzheimer’s disease (AD) is the most prominent cause of dementia [61]. The onset of the disease starts much earlier than the pathological manifestations. AD is characterized by neuron loss, accumulation of amyloid plaques containing amyloid-beta (Aβ) and neurofibrillary tangles containing tau [62].

The involvement of Aβ in RGC apoptosis was demonstrated by Guo et al. in a glaucoma rat model using dark agouti rats. It was found that Aβ plays a role as mediator in pressure induced RGC death, which leads to AMD. The authors studied different strategies to attenuate RGC apoptosis, namely, the use of a β-secretase to reduce Aβ formation, anti-Aβantibody (Aβab) to eliminate Aβ deposits; Congo red to inhibit Aβ aggregation and neurotoxic effects, and a combined therapy with all the treatments. The authors found out that the combination therapy yielded better results than single or dual agent treatments reducing RGC death [63]. Fisichella et al. evidenced the active role of Aβ oligomers in AMD in an in vivo model of AMD on male Sprague-Dawley rats using intravitreally (ITV) injected Aβ oligomers to induce retinal damage. The protective effect of ITV transforming-growth-factor-β1 (TGFβ1), an anti-inflammatory cytokine was also studied. The authors performed a bioinformatics analysis to reveal common gene pathways related to AMD and AD. It was found out that the injection of Aβ oligomers significantly increased the ratio BAX/Blc2, whereas it was significantly prevented using TGFβ1. The coadministration of SB431542, a selective inhibitor of ALK5/TβRI, restrained the effect of TGFβ1, confirming the protective effect of TGFβ1 was wielded through the activation of TGF-β type I receptor (TβRI). Furthermore, the bioinformatics analysis suggested that p-SMAD2 downregulated apoptosis in AMD [64].

Curcumin has been used as a plaque-labeling fluorochrome for the detection of retinal Aβ plaques with the advantage of being detectable earlier than in the brain [65]. Curcumin has also been proposed to have a pleiotropic mechanism of action, targeting on Aβ and tau. It can be intravitreal injected or administered orally, curcumin is capable of crossing the blood brain barrier bound to the beta and tau amyloid plaques and dissolve them, making it an encouraging tool for detection and therapy of AD [66]. Other authors doubt these claims after performing analysis of post-mortem AD human retina and not finding co-staining when using curcumin [67].

## 8. Retinitis Pigmentosa (RP)

Retinitis pigmentosa (RP) is the most frequent hereditary retinal degenerative disorder in adults [68]. It causes a progressive loss of rod photoreceptors followed by a loss of cones [69]. RP is characterized by retinal pigment deposits visible on fundus examination. The most prevalent form of RP is characterized by night blindness, with subsequent loss of peripheral visual field on daylight and ultimately blindness. At the moment there is no therapy that reverts or stops RP progression. The onset of RP starts as soon as the age of two. RP is a heterogeneous group of retinal disorders with autosomal dominant inheritance, autosomal recessive inheritance, or X-linked inheritance [70].

Vasireddy et al. studied the anti-aggregating activity of curcumin in the retina and the recovery of non-functional photoreceptors, as a result of misfolded rhodopsin. Studies were done on COS-7 cell line expressing wild type (wt) or mutant (P23H-R) rhodopsin and on P23H rhodopsin expressing rats. After administration of 100 mg/kg body weight dose of curcumin, the authors assessed that curcumin can cross the blood brain and blood retina barriers when administered orally. Curcumin helped to improve retinal morphology on P23H-R rats, which indicated the preservation of photoreceptors. Levels of expression of rod (rhodopsin and rod outer segment membrane protein 1 (ROM-1)) and cone (S-Opsin and M-Opsin) specific genes were also higher when curcumin was administered. The location of rhodopsin was also reverted to wt after the administration of curcumin [71].

Emoto et al. studied the effect of curcumin against photoreceptor apoptosis induced by the ROS generator methyl-N-nitrosourea (MNU) in a Sprague-Dawley rat model. Doses of 100 or 200 mg/kg curcumin were injected three days prior the MNU administration. Cell survival of the group treated with 200 mg/kg curcumin was statistically significantly better than of the other MNU administered groups. Retinal damage was ameliorated with 200 mg/kg curcumin only in the central retina section. Regarding oxidative DNA damage, levels of 8-hydroxy-2’-deoxyguanosine were quantified and levels on the 200 mg/kg curcumin were similar to the control group, thus suppressing oxidative damage. Lastly, the amount of apoptotic cells after MNU administration was also statistically reduced when curcumin was used [72]. More recently, Scott et al. studied the prenatal protection of curcumin in a Pro-23-His (P23H) rhodopsin mutation swine model. The sows were fed 100 mg/kg body weight/day up to two days prior to parturition. The embryos eyes were enucleated. It was observed that curcumin prevented outer nuclear layer (ONL) and inner nuclear layer (INL) thinning. Curcumin also prevented morphological changes to rod photoreceptors, and as seen on Vasireddy work also helped to prevent misallocated rods [73].

## 9. The Dark Side of the Golden Powder

**Figure 2:**
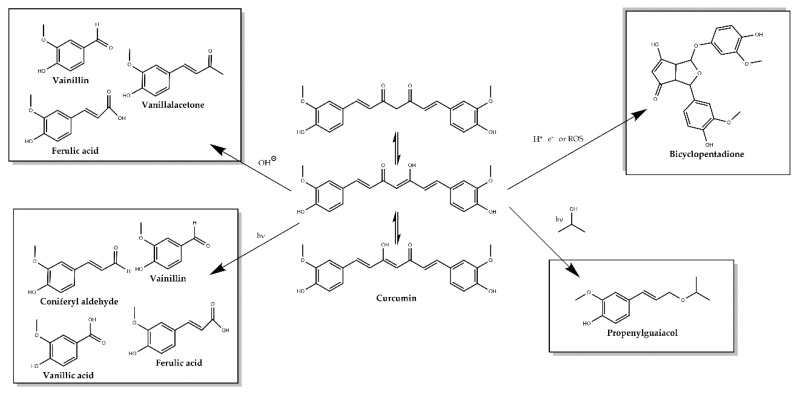
Major degradation products of curcumin under physiological conditions.

Even though the high number of publications describing the many wonders of curcumin, in the last few years, controversy has arisen from different authors classifying curcumin as a PAINS (pan assay interference compounds) and as an IMP (invalid metabolic panacea) [74,75], putting into question many of the findings related to its properties. The main drawbacks found about its use are the extremely low solubility in aqueous medium, the lack of purity of the extracts used on the clinical trials and experiments, having a mixture of compounds labeled as curcumin, while there is a mixture of curcumin, demethoxycurcumin and bisdemethoxycurcumin and other scarcer metabolites [76,77,78]. In addition, under physiological conditions, curcumin rapidly degrades into many different compounds depending on the conditions: ferulic acid, vanillin, ferulic aldehyde and feruloyl methane along other minor products as displayed on Figure 2 [79,80]. All of these facts question which is the actual bioactive product on the proposed effects of curcumin.

## 10. Curcumin Delivery

Different approaches have been attempted during the last few years in order to avoid the first of the aforementioned issues and improve the potential of curcumin as oral administered drug. 

### 10.1. Encapsulation

One of these approaches has been the encapsulation of curcumin with the aim to increase its solubility, stability and bioavailability in physiological conditions [81]. Curcumin has been integrated on different systems, such as folic acid tagged aminated starch/ZnO coated iron oxide nanoparticles [82], curcumin incorporating with Fe_3_O_4_ loaded into: polyethylene glycol–poly lactic acid-co-glycolic acid (PLGA-PEG) co-polymer [83], disulfide-linked hydrophobic backbone of a PEGylated amphiphilic diblock copolymer (biotin poly(ethylene glycol)–poly(curcumin-dithio dipropionic acid)) conjugated with chemotherapeutic agent paclitaxel [84], superparamagnetic iron oxide nanoparticles [85], graphene oxides nanocomposites [86], curcumin-loaded graphene quantum dots [87], cyclodextrin-metal organic frameworks [88], different polymeric nanoparticles like: poly(ethylene glycol)-poly(ε-caprolactone) (PEG-PCL) copolymer [89], PEG-β-cyclodextrin, curcumin solid lipid nanoparticles [90], PCL stabilized with C18-HbPG [91], cholesterol-conjugated poly(D,L-lactide)-based micelles [92] and curcumin-loaded embryonic stem cell exosomes [93]; sequential delivery of curcumin-docosahexaenoic acid loaded carriers towards promoting neuronal survival [94]. We will focus on those applied to retinal pathologies. 

Granata et al. synthesized a micellar nanoaggregate of the calix[4]arene as a nanocarrier for curcumin ocular delivery, which was able to enhance curcumin solubility by 9000 fold and increase its stability 7.5 times; the calix[4]arene nanoaggregate protected curcumin from degradation and also mediated cellular uptake due to the calix[4]arene capability to cross cellular membranes. The effect of the entrapment on curcumin anti-inflammatory and antioxidant properties was assessed in vitro on J774A.1 cell line under lipopolysaccharide (LPS) induced oxidative stress; reducing IκB-α, NF-κB p65 nuclear translocation, COX2 and iNOS expression, to a higher extent compared to free curcumin. A rat model of anterior uveitis, induced by LPS was also evaluated with the same results, the application of curcumin/calix[4]arene decreased ocular inflammation and reduced inflammation proteins to a higher extent than free curcumin. It also reduced ICAM-1 by the suppression of NF-κB [95].

Davis et al. proposed a curcumin nanocarrier comprising TPGS and Pluronic F127, a non-ionic copolymer surfactant, valid as a topical formulation in the treatment of eye diseases, which increased curcumin solubility by a factor of 400,000 and the potential to be transported across ocular barriers; being neuroprotective against glutamate and cobalt chloride induced injury in immortalized retinal cultures in vitro (R28 cells) and preserving the RGCs in ocular hypertension (OHT) and partial optic nerve transection (pONT) murine models [60]. Lim et al. proposed a system using an albumin based nanoformulation in order to increase the solubility of different natural products with low solubility: curcumin, rosmarinic acid (RosA), or ursolic acid (UrsA). Although the antioxidant activity of the different nanoformulations was more efficient for RosA and UrsA when compared to curcumin on ARPE-19 cells under H_2_O_2_ oxidative insult. The authors also performed an ex vivo assay on rabbit corneas and retinas under the same insult to evaluate the protective effects of each nanoformulation; the results showed no signs of tissue damage [96]. The same group proposed the inclusion of curcumin or tetrahydrocurcumin (THC) in hydroxypropyl-γ-cyclodextrin (HP-γCD) and hydroxypropyl-β-cyclodextrin (HP-βCD), which improved water solubility of both drugs, and also studied the drug release rate, with 50% released after 1 h for HP-βCD and almost 2 h for HP-γCD, total drug release was achieved within 8 h in all cases. Regarding cytotoxicity concentrations up to 200 μM did not diminished cell viability in HCE and RPE cells. The inclusion of the drugs was higher on RPE than in HCE and THC showed more permeability than curcumin. Antioxidant activity on both epithelial cells was assessed after an H_2_O_2_ oxidative insult by monitoring SOD1, CAT1 and HMOX1; results showed that the combination of HP-γCD and THC provided higher antioxidant activity and improved bioavailability. An ex vivo assay on rabbit corneas pretreated with the drug inclusion complexes was also performed under H_2_O_2_ oxidative insult; pretreated corneas did not show significant damage. Worth to note, corneas treated with curcumin/cyclodextrin turned yellow whereas THC/cyclodextrin showed no coloration after 12 h, which indicates that the most suitable combination was THC/cyclodextrin [97]. Another proposed carrier was a thermosensitive chitosan-gelatin-based hydrogel containing curcumin-loaded nanoparticles in combination with latanoprost, a drug to increase uveoscleral outflow, in glaucoma treatment. The hydrogel was assayed in human trabecular meshwork cells as a post treatment application after an H_2_O_2_-induced oxidative insult showed reduced levels of inflammation-related gene (TNF, IL-1α, IL-6 and MMP-13), apoptosis, and ROS expression. The hydrogel showed a continuous release profile for 7 days. Biocompatibility was studied in rabbits with no negative effects after the topical use of the hydrogel [98].

### 10.2. Curcumin Analogues

Another approach to improve the stability and the bioavailability of curcumin is the modification of the molecule in order to avoid the problems associated with its oral administration. The poor stability and bioavailability may result from the high reactivity of the β-diketone moiety of its structure [99].

Pittalà et al. designed and studied the effects of nitric oxide-releasing curcumin (VP10/12) and caffeic acid phenethyl ester (CAPE; VP10/39) on oxidative stress caused by H_2_O_2_ oxidative insult in ARPE-19 cells. It was shown that both decreased ROS concentration in a dose dependent manner, being more efficient the CAPE derivative, but not achieving negative control values in either case. It was seen that VP10/12 presented significant cell toxicity at the highest concentration assayed (100 μM). Furthermore, VP10/39 could induce HMOX1 expression [100].

Yet, another proposal by Wang et al. includes the synthesis of the prodrug diphosphorylated curcumin (Cur-2p), and its posterior enzymatic activation, which resulted in a great improvement in terms of stability and a lower aggregation in aqueous media. When administered as intraocular injection, Cur-2p displays good biocompatibility with no morphological or functional alterations of the retina [101]. Using the same approach, Muangnoi et al. synthesized another curcumin prodrug, curcumin diethyl disuccinate (curDD), in order to improve the poor stability of curcumin in physiological pH. The compound was tested on undifferentiated and differentiated ARPE-19 cells. Cell viability was not compromised using up to 10 μM of curDD. Under H_2_O_2_ induced ROS, both curcumin and curDD, when used as pretreatment for 24 h showed protective effects on undifferentiated and differentiated ARPE-19 cells. The molecular mechanism was also evaluated, due to the variations of phosphorylated p44/42, ERK1/2 pathway was proposed. Apoptosis was also inhibited by curcumin and curDD modulating BAX/Bcl2 expression at a transcriptional level. Protein expression of HMOX1 and NAD(P)H quinone dehydrogenase 1 (NQO1) was also increased with curcumin or curDD pretreatment. In all cases curDD also showed a slightly greater protection than curcumin [102].

## 11. Conclusions

There are conspicuous proposed applications to treat and prevent retinal diseases employing curcumin, but still are many loose ends to fully understand its underlying mechanisms. After the feud that took place during the last few years regarding its plausibility as a promising candidate drug, being catalogued as PAINS and IMP; researchers have to be extra-careful on double assessing the results they obtain in order to avoid the above-mentioned problems, and bear in mind the limitations proposed by curcumin critic authors in order to avoid the so-called limitations of the drug.
